# Satiety behavior is regulated by ASI/ASH reciprocal antagonism

**DOI:** 10.1038/s41598-018-24943-6

**Published:** 2018-05-02

**Authors:** Kristen C. Davis, Young-In Choi, Jeongho Kim, Young-Jai You

**Affiliations:** 1 Department of Biochemistry and Molecular Biology, Virginia Commonwealth University, Richmond, VA 23298 USA; 2 Department of Biological Sciences, Inha University, Incheon, 22212 South Korea; 3 Nagoya Neuroscience Institute, Graduate School of Science, Nagoya University, Nagoya, 464-8602, Japan

## Abstract

Appropriate decision-making is essential for ensuring survival; one such decision is whether to eat. Overall metabolic state and the safety of food are the two factors we examined using *C. elegans* to ask whether the metabolic state regulates neuronal activities and corresponding feeding behavior. We monitored the activity of sensory neurons that are activated by nutritious (or appetitive) stimuli (ASI) and aversive stimuli (ASH) in starved vs. well-fed worms during stimuli presentation. Starvation reduces ASH activity to aversive stimuli while increasing ASI activity to nutritious stimuli, showing the responsiveness of each neuron is modulated by overall metabolic state. When we monitored satiety quiescence behavior that reflects the overall metabolic state, ablation of ASI and ASH produce the opposite behavior, showing the two neurons interact to control the decision to eat or not. This circuit provides a simple approach to how neurons handle sensory conflict and reach a decision that is translated to behavior.

## Introduction

Making the correct behavioral choices is essential for any organism’s survival. To make the correct choices, an organism integrates their sensory input to evaluate whether the condition is beneficial or harmful. This holds true from complex organisms such as humans to simpler organisms such as bacteria^1,2^. In nature, an animal often faces conflicting cues and the choice could, in turn, decide the animal’s survival. Behavioral decision-making is influenced heavily by the inputs from sensory neurons and the internal state of the animal^3,4^. In some cases, these decisions are made through sensory integration at the interneuron level^5^. As an example, upon the discovery of a potentially harmful yet nutritious source, an animal must sense its current nutritional status to weigh the nutritional benefit against the potential harm from consuming it. The more starved an animal is, the more risk the animal is willing to take. Determining how these conditions weigh against each other, which neural structures make these calculations, and where the threshold is set for each behavior is a challenging task.

Here we elucidate the *C. elegans* neural circuit that integrates conflicting information given by an aversive (bad) and an appetitive (good) food cue. *C. elegans* have a simple nervous system, consisting of 302 neurons with known connectivity based on a reconstruction of electron micrographs^6^. In addition to a simple, and transparent, nervous system, various optogenetic tools can be used to measure and manipulate the neural activity of a single neuron. Moreover, we previously showed that *C. elegans* exhibits hunger and satiety behaviors similar to those of mammals^7,8^ showing that *C. elegans* is a good genetic model system with conserved behavioral features to study neuro-behavioral mechanisms by which an animal makes its feeding decisions.

We found that the ASI sensory neurons are activated by nutrients (appetitive stimulus), however, this activation is reduced in the presence of an aversive stimulus, which activates the ASH sensory neurons. The ASI neurons promote satiety quiescence that mimics post-prandial sleep in mammals (cessation of movement and feeding following food consumption)^8,9^. The ASH neurons, in contrast, antagonize this satiety quiescence behavior. The antagonistic relationship between ASI and ASH activation by appetitive and aversive stimuli respectively, is modulated by the nutritional status of the animal. Our results identify the neural substrate and its modulation by the animals’ internal state by which conflicting sensory stimuli are processed to result in a behavioral outcome.

## Materials and Methods

### General Methods and Strains

Worms were cultured and handled as described previously^10^ with the following modifications: worms were routinely grown on NGMSR plates, which contain 200 μg/mL streptomycin sulfate, 10 μg/mL nystatin, and 2% instead of 1.7% agar^11^. All worms were maintained at 20 °C on *E. coli* strain HB101^12,13^ unless indicated otherwise. The wild-type strain was *C. elegans* variant Bristol, strain N2^14^. The other strains used were JN1713 *peIs1713*[*sra-6p:: mCasp1*]^15^, CX10979 *kyEx2865*[*sra-6p::GCaMP3, ofm-1::gfp*]^16^, PY7505 *oyIs85*[*gpa-4p::TU813, gcy-27p::TU814, gcy-27p::eGFP, unc-122p::DsRed*]^17^, YJ212 *uyIs212*[*gpa-4p::GCaMP2.2, unc-122::dsRed*] generated from PY6554^17^ integrated via gamma irradiation then outcrossed eight times against N2, YJ216 *uyIs216*[*gpa-4p::GCaMP2.2, unc-122::dsRed*]*; lite-1(ce314); ljIs114*[*Pgpa-13::HFLPase, Psra- 6::HFTFHChR2H YFP*], YJ217 *oyIs84* [*gpa-4p::TU813, gcy-27p::TU814, gcy-27p::eGFP, unc-122p::DsRed*]*; peIs1713* [*sra-6p::mCasp1, unc-122::dsRed*].

### Preparation of Bacteria for Locomotion Assays and Locomotion Monitoring Assays

This assay was conducted as previously described^9^. Briefly, worms were selected as mid to late L4 larvae and cultivated for 8 hours at 20 °C until they reached the young pre-reproductive adult stage. In the fasted condition, these worms were starved on NGMSR plates for 12–14 hours. In the unfasted condition, worms were left on food throughout the assay. Upon reaching the aforementioned adult stage, fasted and unfasted worms were transferred to a 35 mm plate with food and recorded for 1 hour.

### Calcium Imaging

All calcium-imaging experiments were performed on an Axio Observer.A1 with a 63× oil immersion DIC objective and a Hamamatsu C11440 digital camera. Analysis of the imaging data was performed with a custom written program in Mathematica, with one region of interest placed on the neuron of interest and a second placed nearby to measure background.

Young adult worms were picked and placed in a microfluidic device that restrains the worm with the tip of the head (where the ASI and ASH sensory neurons are located) in a stream that can be rapidly switched^18^, “the olfactory chip”). Images were recorded at a rate of 10 frames/second for 60 seconds. Each worm was recorded for a 15 second baseline, followed by exposure to the stimulus for 15 seconds, 15 seconds no stimulus, and a second 15 second exposure to stimulus. During the 15 seconds baseline and the 15 seconds without stimulus, worms were exposed to buffer across their noses. Worms exposed to a treatment during calcium imaging received that treatment for 1–5 minutes prior to the start of the imaging session and received the treatment during the baseline and 15 second period without stimulus.

### Optogenetic Activation of ASH

Worms with channel rhodopsin expressed in ASH were grown their entire lives on either ethanol (21.8 mM) or ethanol containing all trans-retinol (63 µM ATR in 21.8 mM EtOH) and were kept in the dark. Experiments with these worms were performed the same as the other calcium imaging experiments.

### Statistics

All bar graphs denote mean ± SEM. Statistical tests (independent *t*-tests and one-way analysis of variance [ANOVA]) were done using Excel, Prism, MatLab, and Mathematica programming tools. The satiety assay was analyzed using a custom written worm tracking program in MatLab which provides the coordinate data for the centroid of each worm. This data is used to calculate the speed of the worm at various times in the recordings and via a Hidden Markov Model, the percentage of their time spent in the three behavioral states described is determined^9^. Statistical tests for calcium imaging were conducted using data which had been bootstrapped, each set of experiments were randomly resampled up to the *n* available for each set of experiments 1000 times to provide a new, larger sample. For example, if 12 experiments were in the original dataset, the bootstrap randomly selected from this set of experiments for 12 data points and the random selection was performed 1000 times. Bootstrapping was completed to smooth the data and make it possible to compare calcium traces between conditions for statistical testing.

## Results and Discussion

### Aversive stimulus via ASH inhibits ASI activation to nutrients

**Figure 1:**
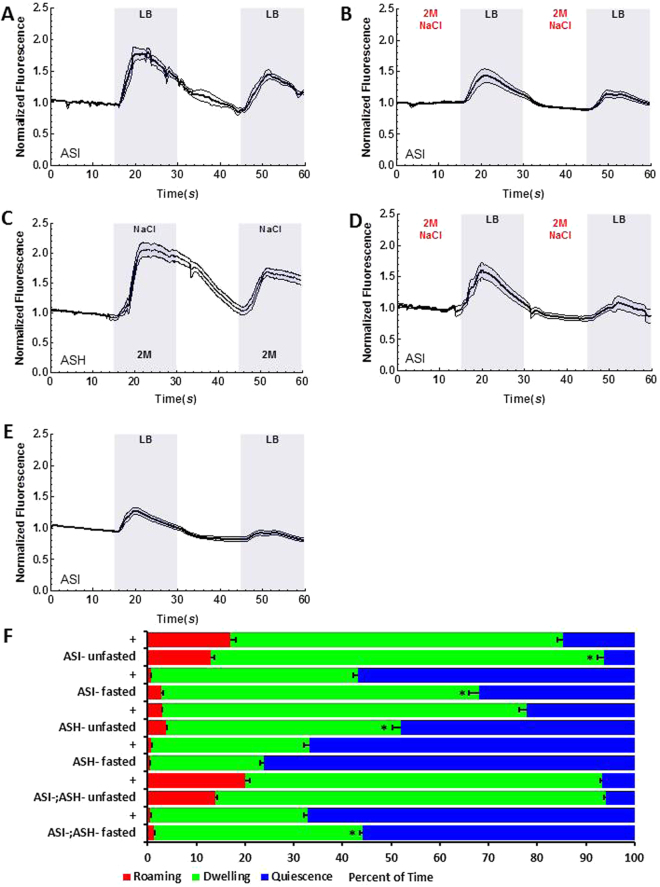
Relationship between ASI & ASH. Graphs A–E are the result of calcium imaging using GCaMP. The gray sections indicate the onset and presence of a stimulus. The white sections indicate buffer flowing across the worm’s nose instead of a stimulus. The darkest line in the traces is the average and the lines above and below the mean are the standard errors of the mean. *Statistically significant (*p* = 0.05). (**A**) Activation to LB in ASI measured using GCaMP (*n* = 17). (**B**) 2 M NaCl suppresses LB activation in ASI of well-fed worms (*n* = 10). (**C**) ASH’s activation to 2 M NaCl. (**D**) Genetic ablation of ASH restores ASI’s LB activation after NaCl (*n* = 10). (**E**) ASI is still capable of LB activation without ASH (*n* = 12). (**F**) The + row of data is the concurrent N2 data for each experiment the + row is above. Significance indicated in this graph is based on a comparison between the concurrently tested N2 and ablation in the row. Satiety quiescence assay results of ASI genetic-ablation fasted (*n* = 7 for ASI^−^, *n* = 16 for ASI^+^) and unfasted (*n* = 11 for ASI^−^, *n* = 20 for ASI^+^), ASH genetic-ablation fasted (*n* = 27 for ASH^−^, *n* = 13 for ASH^+^) and unfasted (*n* = 24 for ASH^−^, *n* = 15 for ASH^+^), and a double genetic-ablation of ASI and ASH fasted (*n* = 27 for ASI^−^ ASH^−^, *n* = 14 for ASI^+^ ASH^+^) and unfasted (*n* = 28 for ASI^−^ ASH^−^, *n* = 14 for ASI^+^ ASH^+^).

**Figure 2:**
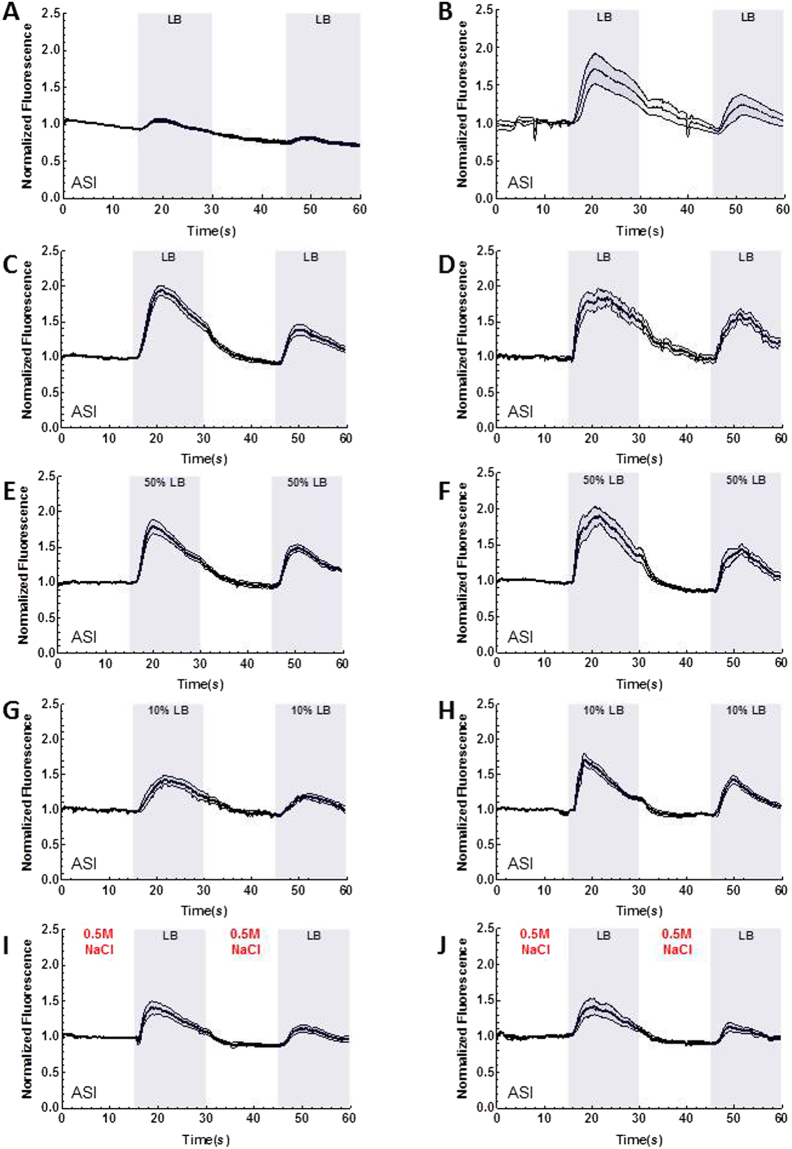
ChR in ASH suppresses ASI and ASI’s activation changes based on nutritional status. Graphs A–J are the result of calcium imaging using GCaMP. The gray sections indicate the onset and presence of a stimulus. The white sections indicate buffer flowing across the worm’s nose instead of a stimulus except in graphs I and J, in these graphs NaCl is flowing across worm’s nose. The darkest line in the traces is the average and the lines above and below the mean are the standard errors of the mean. (**A**) ASH activation by ChR2 decreases ASI’s LB activation (*n* = 10). (**B**) Ethanol control for ChR2 (*n* = 5). (**C**) ASI’s activation to 100% LB under a well-fed condition (*n* = 17). (**D**) ASI’s activation to 100% LB following 6 hours of starvation (*n* = 12). (**E**) ASI’s activation to 50% LB under a well-fed condition (*n* = 10). (**F**) ASI’s activation to 50% LB following 6 hours of starvation (*n* = 10). (**G**) ASI’s activation to 10% LB under a well-fed condition (*n* = 10). (**H**) ASI’s activation to 10% LB following 6 hours of starvation (*n* = 13). (**I**) Suppression of ASI’s activation to LB under a well-fed condition following exposure to 0.5 M NaCl (*n* = 10). (**J**) Suppression of ASI’s activation to LB following 6 hours of starvation and exposure to 0.5 M NaCl (*n* = 11).

ASI are a pair of head sensory neurons in *C. elegans* which are required for satiety behavior and for suppression of food intake. As we showed previously, ASI are activated by Luria Broth (LB), a mixture of nutrients (Fig. 1A)^8^. The LB-induced activation of ASI is reduced by treatment with 2 M NaCl compared to ASI activation in the absence of NaCl (*P* = 0.0002, Fig. 1B), showing that a high concentration of NaCl, an aversive stimulus, inhibits ASI activation by nutrients. This shows that when two conflicting cues exist, the aversive cue suppresses the appetitive cue. As an aversive stimulus induces avoidance in the animal mainly via a pair of head sensory neuron ASH, we hypothesized the ASH are activated by NaCl and this activation in turn inhibits ASI’s activation by nutrients. 2 M NaCl strongly activates ASH (Fig. 1C), showing that NaCl is a proper stimulus for ASH^19^. Next, we tested animals whose ASH are genetically ablated and asked if NaCl suppresses ASI activation to LB in an ASH dependent manner. In worms with ASH genetically ablated, ASI activation to LB is intact (Fig. 1D). Moreover, we see a full restoration of ASI’s response to LB following continuous exposure to NaCl (Fig. 1E), demonstrating ASI inhibition is mediated by ASH. To exclude any potential interference caused by NaCl treatment, we tested the ASI activation by LB after optogenetically activating ASH. When we activated ASH directly and acutely using channel rhodopsin in ASH without any exogenous stimulus, ASI activation to LB is greatly reduced to an extent comparable to continuous exposure to NaCl (Fig. 2A), whereas control animals show normal LB activation (Fig. 2B). This result shows ASH is sufficient to inhibit ASI activation to LB. Therefore, we conclude that ASI activation to LB, an appetitive cue, is inhibited by ASH, which are activated by an aversive stimulus.

### ASI and ASH interact to regulate satiety behavior

To understand the behavioral implications of the interaction between ASI and ASH neurons, we examined satiety quiescence of animals in which the two neuron pairs were genetically ablated individually and in combination. We measured satiety behavior using the automated system we built^9^. Briefly, a user-built 9 camera monitoring system records individual animal’s locomotion. Then the data is analyzed by a Hidden Markov Model to measure the percentage of time the animal spends in each behavioral state: the three behavior states are roaming (searching for food), dwelling (browsing current food source), and satiety quiescence (not eating or moving)^9^. After starvation and refeeding, conditions under which an animal shows the most consistent and robust satiety quiescence, animals lacking ASI show increased dwelling behavior in comparison to concurrent controls (Fig. 1F, + and ASI- unfasted) as shown in our previous work. This confirms that ASI is required for switching from dwelling to quiescent behavior^8^. Conversely, animals lacking ASH show increased satiety quiescence in both fasted and unfasted conditions (Fig. 1G, + and ASH- unfasted). The removal of ASH increases the time spent in quiescence and showed opposite behaviors to that of the removal of ASI. This suggests ASH is required for switching from quiescence to dwelling and by removing this signal from ASH, the potential signal for suppression of quiescent behavior could be alleviated, resulting in an increase of time spent in quiescence. The animals lacking both pairs of neurons most closely resemble the worms lacking ASI, indicating ASI is downstream of ASH for satiety quiescence when an animal is well-fed. This is consistent with the calcium imaging data where ASH controls ASI acting upstream. Taken together, ASH inhibits ASI both in neuronal activity and the relevant feeding behavior when the animal is well-fed.

### ASI-ASH interaction depends on the animal’s nutritional status

Next we asked whether ASI activation by nutrients is altered by the animal’s nutritional status. When we measured ASI activation to different concentrations of LB, lower concentrations of LB produce progressively smaller activation in ASI for well-fed worms (Fig. 2C,E,G). ASI activity after 6 hours of starvation was also reduced by lower concentrations of LB (Fig. 2D,F,H). However at 10% LB, the activation is significantly larger in starved animals compared to well-fed worms (area under the curve and peak of activation, *P* < 0.0001, Fig. 2G,H). This suggests that ASI activity is modulated by the animal’s nutritional status. Thus, we decided to test the interaction between ASI and ASH in a starved condition. There are two possible outcomes: (a) during starvation, ASI could override the signal from ASH to respond normally to the nutrient-rich stimulus or (b) ASH could override the signal from ASI to respond normally to the aversive stimulus. To determine if nutritional depletion could overcome the aversive stimulus’ suppression of ASI, we fasted worms for 6 hours and tested them using continuous exposure to 0.5 M NaCl followed by the presentation of LB. There was no significant difference between well-fed worms and fasted worms in these stimulus conditions (Fig. 2I,J). The aversive stimulus may be too strong to overcome with only 6 hours of starvation; however a longer time in starvation makes the worms more fragile and difficult to load into the microfluidic device.

**Figure 3:**
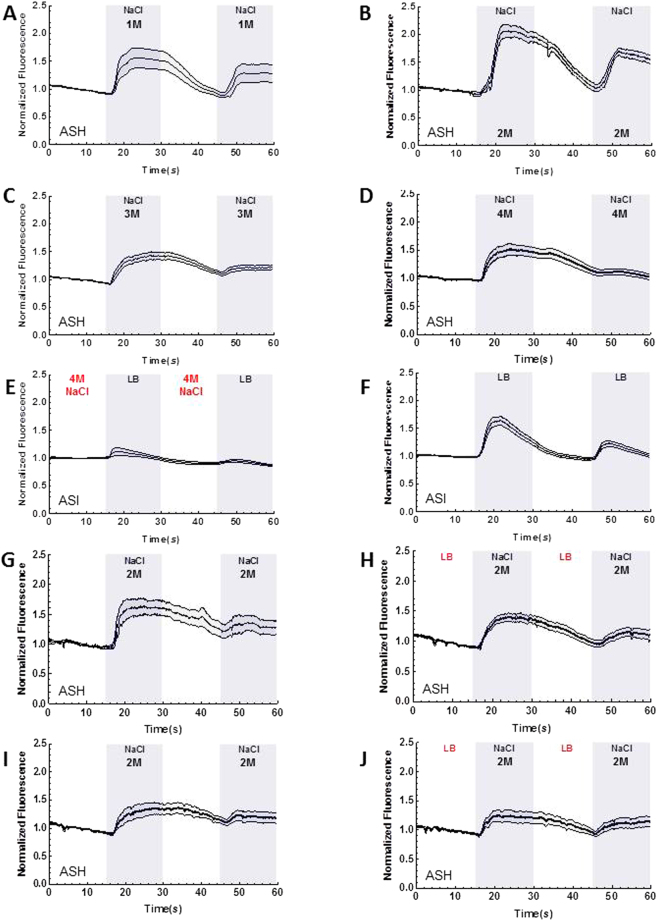
ASH activation is influenced by feeding status. Graphs A–H are the result of calcium imaging using GCaMP. The gray sections indicate the onset and presence of a stimulus. The white sections indicate buffer flowing across the worm’s nose instead of a stimulus except in graphs E and H. The darkest line in the traces is the average and the lines above and below the mean are the standard errors of the mean. (**A**) ASH’s activation to 1 M NaCl (*n* = 13). (**B**) ASH’s activation to 2 M NaCl *This is the same data shown in Fig. 1C, it is included here for comparison to the other NaCl concentrations (*n* = 12). (**C**) ASH’s activation to 3 M NaCl (*n* = 13). (**D**) ASH’s activation to 4 M NaCl (*n* = 14). (**E**) Suppression of ASI’s activation to LB following exposure to 4 M NaCl (*n* = 12). (**F**) ASI’s activation to LB before or after (alternating conditions) experiments with 4 M NaCl (*n* = 14). (**G**) ASH’s activation to 2 M NaCl following 6 hours of starvation (*n* = 7). (**H**) Suppression of ASH’s activation to 2 M NaCl following exposure to LB and 6 hours of starvation (*n* = 10). (**I**) ASH’s activation to 2 M NaCl (*n* = 10), same day control for J. (**J**) ASH’s activation to 2 M NaCl after exposure to LB (*n* = 11).

Next we tested whether the nutritional status also influences ASH activation by the aversive stimulus. We hypothesized that if the animals are starved, the response to the aversive stimulus could be altered in the presence of a nutritious cue. This could be relevant for a starved animal in the wild that encounters potentially dangerous food. Reduction of ASH activity to the dangerous cue in the presence of a nutritious cue could allow the animal to take the risk of eating the harmful food. All four concentrations we tested (1, 2, 3, 4 M NaCl) activated ASH in the well-fed animals (Fig. 3A–D), the highest level of activation was seen with 2 M NaCl (Fig. 3B). This could be because 3 M or 4 M NaCl might inhibit the neuronal activity even if 4 M NaCl does not kill the neurons; when we washed off the NaCl, the animal showed an intact response to LB, showing that ASI were not damaged and still capable of responding to stimuli (Fig. 3E,F). After 6 hours of fasting, ASH activation to 2 M NaCl is partially suppressed by LB (Fig. 3H) in comparison to ASH activation following 6 hours of fasting in the absence of LB (Fig. 3G) (area under the curve, insignificant *P* = 0.098). Even if the suppression does not reach significance (Fig. 3H), this suppression is not seen in the well-fed condition when we compared NaCl activation in ASH (Fig. 3I) with NaCl activation in ASH following LB exposure (Fig. 3J). This suggests the increased responsiveness of ASI to a nutritious cue by starvation might negatively influence ASH’s activity, yet our starvation and imaging conditions may not be sufficient to elicit a calcium-based response that is statistically significant.

Considering our work and the work of others examining the interaction between ASI and ASH^20^, there is a reciprocal antagonism between ASI and ASH which could be regulated by the metabolic state. In this model, metabolic state switches the hierarchy of the interaction so that the animals prioritize their choice; hungry animals with reduced ASH and enhanced ASI responsiveness could take the risk of eating food with an aversive cue, while satiated animals with enhanced ASH and reduced ASI responsiveness would avoid it.

**Figure 4:**
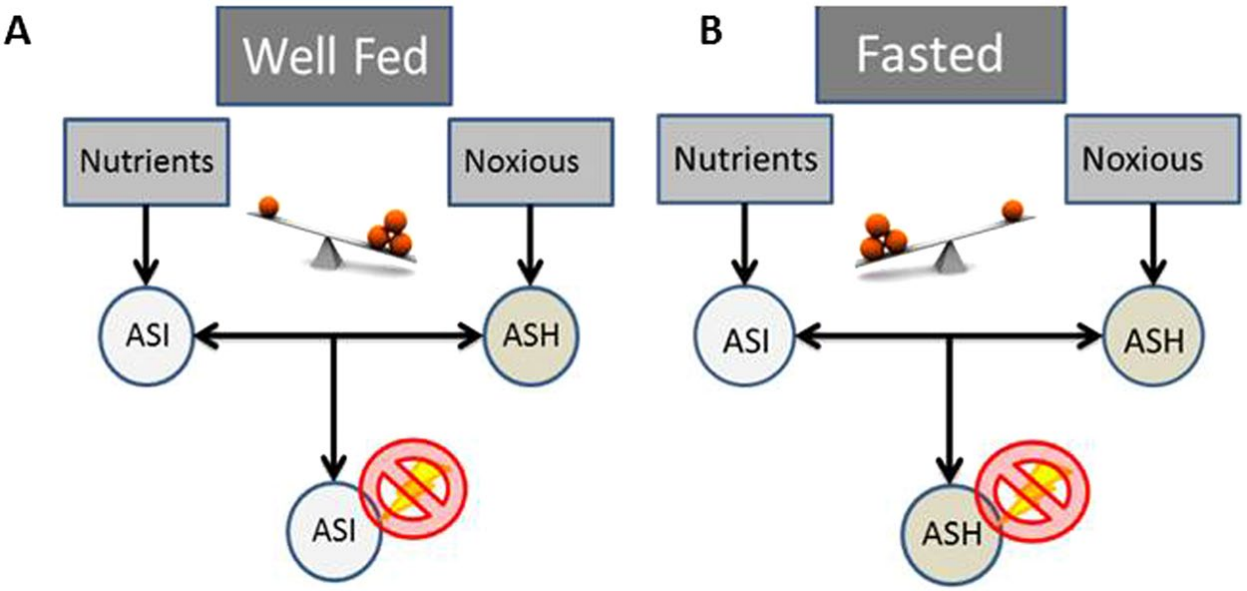
Proposed model for ASI and ASH activity influenced by internal and external cues. (**A**) Under well-fed conditions the balance between ASI and ASH favors the activity of ASH when nutrients and a noxious stimulus are present. (**B**) During fasting conditions the balance between ASI and ASH favors the activity of ASI when nutrients and a noxious stimulus are present.

Previous work has shown reciprocal suppression of ASI and ASH in the context of heavy metal chemotaxis^20^ but not in the context of feeding behavior or aversive and nutritious stimuli. We suggest that our work could further support the importance of this circuit in decision-making that is relevant to the essential choice of feeding based on nutritional status. Based on our results, we suggest that under well-fed conditions, ASH are capable of suppressing ASI’s activation to nutrients (Fig. 4A). However, under certain conditions, ASI might be able to suppress ASH’s activation to aversive stimuli^20^. This crosstalk between two neurons will enhance or suppress feeding behaviors based on the integration of internal and environmental cues. We speculate that this integration could be mediated by a set of interneurons acting as a sensory integrator between these two opposing signals. Previous work has demonstrated an interneuron pair, AIA, are involved in a behavioral choice between diacetyl (appetitive stimulus) and copper (aversive stimulus) by changing its expression profiles^5^. The ASI-ASH crosstalk could incorporate the integrating function of AIA to make behavioral decisions regarding feeding. Further work would be required to reach this conclusion without speculation.
